# Supplementary material for: Mechanisms governing the pioneering and redistribution capabilities of the non-classical pioneer PU.1
Source: Nat Commun. 2020 Jan 21;11:402. doi: 10.1038/s41467-019-13960-2 (PMC6972792; doi:10.1038/s41467-019-13960-2)
Supplement: Supplementary file 7 — Source data [file 41467_2019_13960_MOESM7_ESM.zip › Source_Data/FigureS2/FigureS2B_MotifScanOutput/KmeansCluster13/homerResults/motif1.similar.html]

motif1

## Information for motif1

T
C
A
G
C
T
G
A
T
A
C
G
T
G
C
A
A
C
T
G
A
C
T
G
C
G
T
A
C
G
T
A
T
C
A
G
A
G
C
T
T
A
C
G
T
C
A
G
  
Reverse Opposite:  

A
G
T
C
A
T
G
C
T
C
G
A
A
G
T
C
C
G
A
T
A
C
G
T
A
G
T
C
A
G
T
C
A
C
G
T
A
T
G
C
G
A
C
T
A
G
T
C
  

|  |  |
| --- | --- |
| p-value: | 1e-608 |
| log p-value: | -1.402e+03 |
| Information Content per bp: | 1.630 |
| Number of Target Sequences with motif | 1242.0 |
| Percentage of Target Sequences with motif | 73.40% |
| Number of Background Sequences with motif | 6589.2 |
| Percentage of Background Sequences with motif | 14.61% |
| Average Position of motif in Targets | 101.9 +/- 37.6bp |
| Average Position of motif in Background | 100.5 +/- 69.7bp |
| Strand Bias (log2 ratio + to - strand density) | 0.0 |
| Multiplicity (# of sites on avg that occur together) | 1.36 |
| Motif File: | file (matrix) reverse opposite |

### Similar de novo motifs found

|  |  |  |  |  |  |  |  |
| --- | --- | --- | --- | --- | --- | --- | --- |
| Rank | Match Score | Redundant Motif | P-value | log P-value | % of Targets | % of Background | Motif file |
| 1 | 0.986 | T C G A T C G A T A C G T G C A A C T G A T C G G T C A G C T A T C A G G A C T A T C G | 1e-596 | -1372.752703 | 74.70% | 15.84% | motif file (matrix) |
| 2 | 0.974 | T A G C C T G A A T G C C G A T A C G T A G T C A G T C A C G T A G T C G A C T | 1e-590 | -1360.023317 | 66.78% | 11.42% | motif file (matrix) |
| 3 | 0.979 | A G T C T A G C C T G A A G T C C G A T A G C T A G T C A G T C A C G T A T G C G A C T A G T C A G C T | 1e-569 | -1312.158108 | 76.48% | 17.96% | motif file (matrix) |
| 4 | 0.964 | A G T C A T G C C T G A A T G C G C A T G A C T T G A C T A G C A C G T | 1e-529 | -1219.551430 | 75.53% | 18.84% | motif file (matrix) |
| 5 | 0.981 | T C A G C T A G T C A G C T G A T A C G T G C A A C T G A C T G C G T A C G T A C T A G A G C T A T C G T C A G | 1e-525 | -1208.897308 | 60.11% | 9.87% | motif file (matrix) |
| 6 | 0.939 | T G C A A C T G A T C G C G T A C G T A T A C G A C G T T A C G | 1e-477 | -1100.564116 | 59.87% | 11.10% | motif file (matrix) |
| 7 | 0.920 | G T A C T C G A A G T C A C G T A C G T A G T C A T G C A C T G A T C G G A C T A T C G | 1e-451 | -1040.406914 | 72.16% | 19.65% | motif file (matrix) |
| 8 | 0.922 | A C T G A C T G C G T A C G T A A C T G A C G T A T C G | 1e-380 | -876.401895 | 50.35% | 9.33% | motif file (matrix) |
| 9 | 0.905 | C T G A T C G A T C G A T A C G T G A C A T C G C T A G C T G A C T G A T A C G | 1e-372 | -857.490661 | 72.70% | 23.94% | motif file (matrix) |
| 10 | 0.881 | A T G C G C A T C G A T G A T C A T G C A C T G A T G C G A C T G A C T | 1e-371 | -854.297956 | 82.15% | 32.57% | motif file (matrix) |
| 11 | 0.866 | G T A C A C T G A C T G C G T A C G T A T C A G G A T C T C A G | 1e-350 | -807.063775 | 80.20% | 31.93% | motif file (matrix) |
| 12 | 0.860 | A T G C T G A C G T C A C A G T A T G C A C G T A C G T A T G C A G T C A C G T A G T C G C A T G T C A G A C T | 1e-305 | -703.391254 | 74.05% | 29.09% | motif file (matrix) |
| 13 | 0.870 | A T G C C G A T A C G T A G T C A G T C A C T G A T G C G A C T | 1e-304 | -700.851452 | 67.02% | 23.24% | motif file (matrix) |
| 14 | 0.850 | A T G C A G C T C G A T G T A C G T A C A C T G A G T C | 1e-267 | -615.878435 | 72.46% | 30.25% | motif file (matrix) |
| 15 | 0.863 | G A C T A G T C C T G A A G T C C G A T C G A T A G T C T G A C G A C T G T A C | 1e-261 | -602.495671 | 54.26% | 16.44% | motif file (matrix) |
| 16 | 0.803 | C G A T C G A T A G T C A T G C A C T G A T C G G A C T | 1e-239 | -552.047856 | 57.21% | 19.73% | motif file (matrix) |
| 17 | 0.888 | A G T C T C G A A G T C C G A T A C G T A T G C A T G C A C T G C T A G C G A T | 1e-239 | -551.980658 | 41.19% | 9.69% | motif file (matrix) |
| 18 | 0.873 | A C T G A T G C T C G A A G T C C G A T A C G T A G T C A T G C A C T G C T A G A G C T T A C G | 1e-188 | -433.029599 | 48.76% | 17.01% | motif file (matrix) |
| 19 | 0.770 | C G T A A T C G A G C T G A C T A T G C G A T C C A G T G A T C G T A C A G T C G A T C A G T C | 1e-187 | -432.307262 | 54.20% | 20.95% | motif file (matrix) |
| 20 | 0.761 | A T G C C G A T C G A T A G T C A G T C A C T G A T C G | 1e-153 | -353.034750 | 39.36% | 12.99% | motif file (matrix) |
| 21 | 0.865 | T C A G A T C G A G T C T G C A A T G C C G A T C A G T A T G C A G T C T A C G A C G T A C G T C G A T T A C G | 1e-110 | -253.643911 | 23.05% | 5.98% | motif file (matrix) |
| 22 | 0.824 | G T A C T C A G C G T A C G T A A C T G A C T G C G T A C T G A A T C G A G C T A T C G C A G T | 1e-67 | -154.953448 | 13.00% | 3.01% | motif file (matrix) |
| 23 | 0.791 | A G T C C T G A A C G T A C G T A C G T A G T C A G T C | 1e-65 | -151.822298 | 24.94% | 9.97% | motif file (matrix) |
| 24 | 0.784 | T A G C T G C A A T G C G C A T C G A T G T A C A T G C A T G C | 1e-62 | -142.836355 | 17.43% | 5.61% | motif file (matrix) |
| 25 | 0.775 | A T G C A T G C G T C A A T G C G A C T A G C T G A T C A G T C G A C T T A G C G C A T A C T G | 1e-54 | -124.758217 | 10.46% | 2.40% | motif file (matrix) |
| 26 | 0.763 | T A G C C G T A G T A C A G C T C A G T A T G C G A T C A T C G G T A C C A G T A C G T | 1e-34 | -78.641381 | 8.92% | 2.66% | motif file (matrix) |
